# T-Cell Adhesion in Healthy and Inflamed Skin

**DOI:** 10.1016/j.xjidi.2021.100014

**Published:** 2021-04-30

**Authors:** Joshua M. Moreau, Victoire Gouirand, Michael D. Rosenblum

**Affiliations:** 1 Department of Dermatology, University of California San Francisco, San Francisco, California, USA

**Keywords:** AD, atopic dermatitis, BM, basement membrane, DC, dendritic cell, DETC, dendritic epidermal γδ T cell, ECM, extracellular matrix, HF, hair follicle, JC, John Cunningham, LAD, leukocyte adhesion deficiency, PML, progressive multifocal leukoencephalopathy, Th, T helper, Treg, regulatory T cell, Trm, tissue-resident memory

## Abstract

The diverse populations of tissue-resident and transitory T cells present in the skin share a common functional need to enter, traverse, and interact with their environment. These processes are largely dependent on the regulated expression of adhesion molecules, such as selectins and integrins, which mediate bidirectional interactions between immune cells and skin stroma. Dysregulation and engagement of adhesion pathways contribute to ectopic T-cell activity in tissues, leading to the initiation and/or exacerbation of chronic inflammation. In this paper, we review how the molecular interactions supported by adhesion pathways contribute to T-cell dynamics and function in the skin. A comprehensive understanding of the molecular mechanisms underpinning T-cell adhesion in inflammatory skin disorders will facilitate the development of novel tissue-specific therapeutic strategies.

### Overview

Adhesion is a fundamental process in immune cell function. This is definitively demonstrated by leukocyte adhesion deficiency (LAD) syndrome, a rare genetic disorder caused by defects in a select number of adhesion genes, especially in *ITGB2*. Without bone marrow transplantation, patients with severe disease are prone to recurrent infections, demonstrate impaired wound healing, and are unlikely to survive beyond 2 years of life (Almarza Novoa et al., 2018). Immune responses in peripheral tissues require adhesion pathways to mediate cell recruitment, tissue immunosurveillance, and intercellular interaction between different cell types. These activities are dependent on a large assemblage of adhesion proteins, including integrins, selectins, and cadherins that mediate cell‒cell and cell‒extracellular matrix (ECM) interactions. Contact between adhesion molecules and their ligands provides a means for force transmission and signal transduction that enables cellular movement, positioning, and the tight interactions required for immune synapse formation (Basu and Huse, 2017; Cavallaro and Dejana, 2011; Hammer et al., 2019). Adhesive function is directly driven by adhesion proteins but also encompasses the layers of regulatory mechanisms that create cell lineage (i.e.*,* CD4^+^ T cell vs. CD8^+^ T cell), cell state (i.e.*,* quiescence vs. activation), and tissue (i.e.*,* skin vs. colon) specificity.

Mammalian skin contains large populations of tissue-resident and recirculating T cells. These cells protect against infection and also play an important role in maintaining skin barrier integrity and tissue homeostasis. Regulatory T cells (Tregs) interact with epithelial stem cells to facilitate differentiation during hair follicle (HF) cycling (Ali et al., 2017b)**,** whereas Tregs, subsets of CD8^+^ T cells, MAIT cells, and γδ T cells contribute to wound repair and re-epithelialization after skin injury (Constantinides et al., 2019; Harrison et al., 2019; Jameson et al., 2002; Nosbaum et al., 2016). In addition, Tregs keep local immune responses in balance by tightly regulating inflammatory signaling (Kalekar and Rosenblum, 2019; Kalekar et al., 2019). Performing these functions in the skin requires adhesion-mediated recruitment, interstitial migration, and cell‒cell interaction. In this study, we follow T cells on a journey as they enter and traverse the skin, interact with antigen-presenting cells or malignant cells, and eventually emigrate to draining lymph nodes. We review how the outcome of this journey is determined by the composition of adhesion molecules expressed on various skin T-cell populations and describe how inflammation and immune activation shape adhesive capacity.

Not surprisingly, dysregulation of T cells in the skin is a prominent feature of many skin disorders, and either augmentation or suppression of their activity provides a compelling strategy toward treating human disease (Ho and Kupper, 2019). Given the importance of cellular adhesion in immune cell function, adhesion molecules have long been considered promising therapeutic targets (Ley et al., 2016). Research and clinical efforts in this area have produced several therapies currently approved for the treatment of autoimmune diseases, including multiple sclerosis and Crohn’s disease (Ley et al., 2016). Unfortunately, these successes have yet to be realized for the treatment of skin disorders. To date, Efalizumab, the only adhesion-targeting therapy approved for a skin disease (psoriasis) was withdrawn from clinical use secondary to severe and potentially life-threatening side effects (Pugashetti and Koo, 2009). Nonetheless, rapid progress in understanding the nuances surrounding T-cell adhesion in tissues provides the justification for continued exploration of these pathways as targets for therapeutic manipulation. In this paper, we discuss the attempts to develop therapies modulating T-cell adhesion and examine the opportunities for applying new research findings toward developing treatments for skin disease.

### A T cell’s guide to skin adhesion

In the absence of inflammatory signals, tissue-resident T cells, including tissue-resident memory (Trm) cells and dendritic epidermal γδ T cells (DETCs) (in mice), continually survey the skin while other subsets circulate between the tissue, draining lymph nodes, and peripheral blood (Brown et al., 2010; Collins et al., 2016; Gebhardt et al., 2011). Despite the significant differences in the ontogeny and transcriptional signature of tissue-resident and recirculating T cells (Szabo et al., 2019), adhesion molecules are a shared molecular requirement for movement into and through the skin. However, differential induction or activation of adhesion pathways contributes to nuances in the functional behaviors of each population. Broadly, there are four phases of T-cell behavior dependent on adhesion: entry into the skin, interstitial migration, interactions with other cells, and egress from the skin (Figure 1). An individual T cell may not be primed to perform each step sequentially, but it will need to express a specific set of molecules to accomplish any given task. In the following sections, we discuss how T cells use adhesion molecules in the context of each process. However, it should be noted that the majority of research in this field to date has focused on proinflammatory T cells (i.e., conventional CD4^+^ and CD8^+^ T cells) and that much less is known about how Tregs utilize adhesion and adhesion-associated molecules to regulate their functions in the skin.

**Figure 1:**
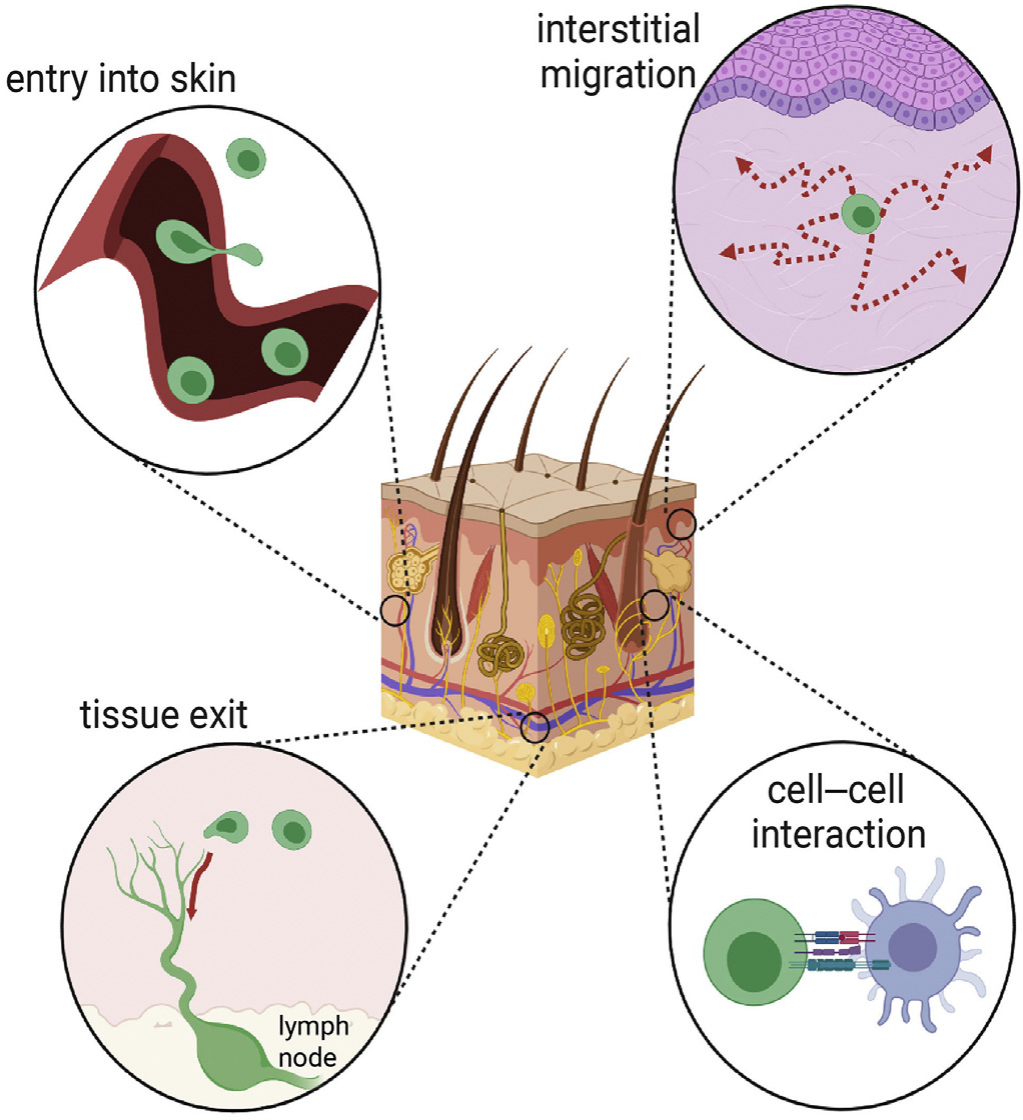
**Cellular adhesion is required for T-cell function in the skin.** To extend immune protection to peripheral tissues, T cells must actively leave circulation, migrate through interstitial spaces, interact with other cells, and eventually return to circulation by lymphatic vessels. Each of these processes is dependent on adhesive interactions between T cells and other cells or between T cells and the extracellular matrix. Expression patterns of adhesion molecules temporally and spatially regulate T-cell dynamics by selectively assisting specific movements. Only those T cells expressing the correct collection of adhesion receptors will be able to traverse the tissue environment. Inflammation controls T-cell access to the tissue by modulating this adhesion molecule signature.

#### A sticky toolkit: regulation of T-cell adhesion

Cellular adhesion is principally driven by several groups of molecules expressed on T cells, including integrins, selectins, cadherins, and Ig superfamily members (Figure 2) (Harjunpää et al., 2019). T cells also express a number of other glycoproteins, such as CD44, that contribute to mediating adhesion (Baaten et al., 2012). Many additional proteins indirectly influence cellular behavior by regulating the ability of expressed adhesion molecules to bind their ligand. For example, most integrins are present on the cell surface in a relatively closed conformational state with a low affinity for ligand binding. TCR engagement or chemokine exposure produces signaling cascades that induce a conformational change in both integrin subunits. This extends the integrin into an active form and substantially increases ligand affinity (Harjunpää et al., 2019). Transformation into an active conformation is dependent on the integrin-binding proteins, talin and kindlin, which mediate force transmission through connections to the cytoskeleton (Harjunpää et al., 2019; Sun et al., 2019). Through these cytoskeletal interactions, integrins and other adhesion molecules impart T cells with mechanosensitivity that is essential for navigating the mechanically dynamic tissue environment of the skin (Biggs et al., 2020; Sun et al., 2019).

**Figure 2:**
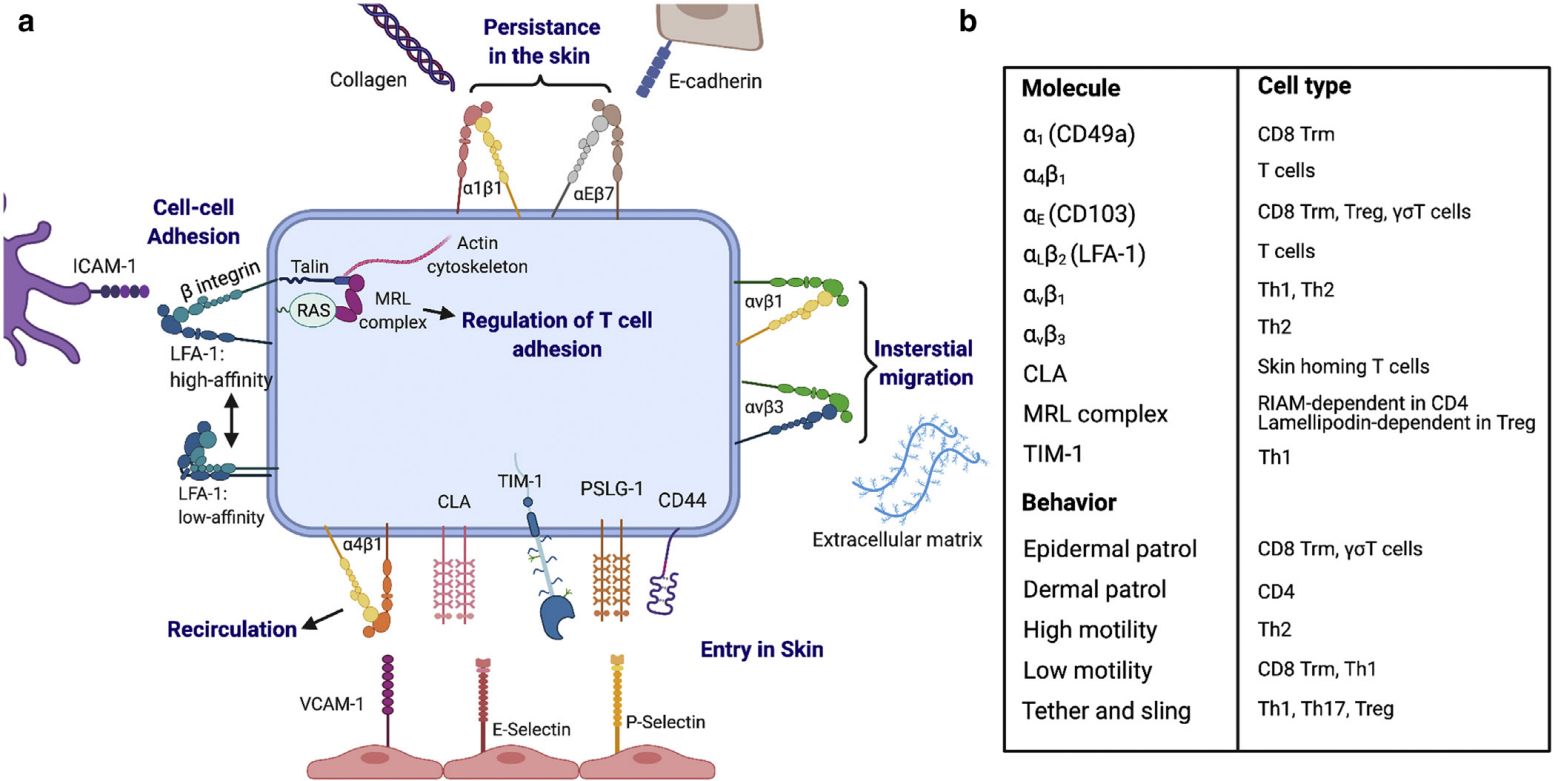
**A diverse set of molecules drive T-cell adhesion.** (**a**) To enter the skin, T cells must interact with blood vessel endothelial cells (**a**, bottom) before crossing the endothelium into the tissue parenchyma. Initial tethering and/or rolling interactions are mediated by low-affinity binding between the T-cell glycoproteins PSLG-1, CLA (a carbohydrate variant of PSLG-1), and CD44, with E- and P-selectin expressed on the endothelium. Firm adhesion and transendothelial migration require high-affinity ligation of the integrins LFA-1 and VLA-4 with the Ig superfamily members ICAM-1 and VCAM-1. Once in the skin, interactions between T cells and skin epithelium as well as the interaction of T cells with infected or cancerous target cells are also integrin dependent (**a**, top and left). Integrin α_E_β_7_ binding to E-cadherin is especially important for association with the epithelium. (**b**) Although the expression of cell adhesion molecules is highly sensitive to the inflammatory milieu, several differences in adhesion activity have been observed across T-cell subsets. Heterogeneity in the levels and timing of adhesion molecule expression will directly influence T-cell behavior in the skin, including tissue entry, immunosurveillance, and local migration patterns. Th, T helper; Treg, regulatory T cell; Trm, tissue-resident memory.

Interestingly, additional adaptor proteins modulate talin‒integrin interactions leading to cell lineage and cell state‒dependent augmentation of adhesion. Recently, we found that the talin-binding C-type lectin, layilin, is preferentially expressed on highly activated CD8^+^ T cells infiltrating melanoma tumors but is nearly absent on T cells in peripheral blood (Borowsky and Hynes, 1998; Mahuron et al., 2020). Layilin enhances integrin α_L_β_2_ (LFA-1) activation and binding to its ligand ICAM-1 to promote antitumor immunity (Mahuron et al., 2020). CD8^+^ T cells expressing high levels of IL-17 (Tc17 cells) infiltrating psoriatic lesions are also enriched for layilin expression compared with T cells from healthy skin, suggesting that although layilin may protect against cancer, it could contribute to autoimmunity (Liu et al., 2020). Differential regulation of integrin activation was also demonstrated between Tregs and CD4^+^ T-conventional cells. Whereas most CD4^+^ T cells are entirely dependent on RIAM to mediate β integrin binding to talin, this protein is dispensable in Tregs. Deletion of RIAM protected mice in a spontaneous colitis model owing to a reduced accumulation of effector T cells in the gut, whereas Treg homing to this tissue was normal (Sun et al., 2021). Lamellipodin, a RIAM paralog preferentially expressed in Tregs, is able to facilitate integrin‒talin binding in these cells (Sun et al., 2021).

The expression pattern of adhesion molecules themselves also has importance in dictating T-cell function. For T cells to gain access to peripheral tissues, they must express an adhesion signature appropriate for that tissue. Both γδ T cells and αβ T cells require the expression of the glycoprotein CLA to facilitate skin entry by binding to E-selectin expressed on skin endothelial cells (Berg et al., 1991; Biedermann et al., 2002; Fuhlbrigge et al., 1997; Jiang et al., 2010). Studies examining T-cell recruitment under inflammatory conditions have implicated CD43 and CD44 as additional E-selectin ligands contributing to skin entry (Ali et al., 2017a; Nácher et al., 2011). Integrin expression is also critical because CD18^−/−^ (the β_2_ subunit of LFA-1) knockout mice exhibited reduced inflammation in allergic contact dermatitis and delayed-type hypersensitivity reactions owing to impaired T-cell accumulation in skin lesions. Notably, Langerhans cell precursors and dendritic cells (DCs) displayed normal migratory behavior (Grabbe et al., 2002). Conversely, mice with a hypomorphic mutation that reduces CD18 levels to 2–16% of wild type spontaneously develop skin lesions mirroring human psoriasis (Bullard et al., 1996). In this context, both CD4^+^ and CD8^+^ T cells efficiently enter the skin, and tissue pathology is potentiated by a defective Treg compartment (Kess et al., 2003; Singh et al., 2013; Wang et al., 2008). Reduced CD18 was observed to hinder Treg interaction with DCs, impair their suppressive capability, and promote lineage instability by converting Tregs to IL-17‒producing effector T helper (Th)17 cells (Singh et al., 2013; Wang et al., 2008). Another integrin important for skin-specific function (although not for entry into the skin) is α_E_β_7_. CD103 (the α_E_ subunit of α_E_β_7_) is a marker of Trm cells and is widely expressed among T cells in the skin (Hardenberg et al., 2018). α_E_β_7_ interacts with E-cadherin and likely has a role in positioning T cells within the tissue by allowing an interaction with the epithelium (discussed in detail in the following section) (Hardenberg et al., 2018). Mice deficient in CD103 develop severe skin lesions at least partially owing to a defective Treg compartment (Braun et al., 2015; Schön et al., 2000). Collectively, these observations begin to define an adhesome signature intrinsically connected to T-cell function in the skin.

#### Entry into the skin

T-cell entry into the skin is initiated by tethering and rolling interactions with blood vessel endothelial cells (Garrood et al., 2006; Schön et al., 2003). CLA and a related glycoprotein, PSLG-1 (CD162), expressed on T cells bind endothelial E- and P-selectin. In addition, CD44 coassociation with integrin α_4_β_1_ (VLA-4) has been found to facilitate early binding to the endothelial ligand VCAM-1 (Issekutz and Issekutz, 2002; Nandi et al., 2004; Schön et al., 2003; Siegelman et al., 2000). These relatively low-affinity interactions act to slow down the T cells caught in high-speed blood flow (Garrood et al., 2006; Schön et al., 2003). Some nuance has been reported in the mechanisms utilized by different T-cell subsets to initiate tethering. Th1, Th17, and Tregs were found to use a tether and sling behavior to improve adherence efficiency. These subsets were markedly better at forming these structures than Th2 cells, which directly translated into superior attachment to the vessel wall (Abadier et al., 2017). Similarly, in Th1 and Th17 but not in Th2 cells, TIM-1 binds P-selectin to promote skin entry (Angiari et al., 2014). Interestingly, this may explain why tissue-resident Th2 cells are absent in healthy skin of C57BL/6 mice, whereas Tregs and Th1 cells are readily observed (Nussbaum et al., 2013).

Low-velocity travel along vessel walls stimulates integrin activation leading to firm adhesion primarily through LFA-1/ICAM-1 and VLA-4/VCAM-1 binding (Garrood et al., 2006; Schön et al., 2003). Firm adhesion fully arrests the T cells and allows cytoskeleton reorganization and cellular polarization to form a leading-edge lamellipodium. Lymphocytes then traverse the endothelium primarily through endothelial cell junctions (Garrood et al., 2006; Sumagin and Sarelius, 2010). Integrins facilitate transendothelial migration through bidirectional signaling that both increases endothelial permissiveness and directly prepares T cells for tissue entry. LFA-1 engagement with ICAM-1 on endothelial cells induces a MAPK signaling cascade that in turn promotes vascular permeability and induces cytokine and chemokine production (Dragoni et al., 2017; Sumagin and Sarelius, 2010). In T cells, LFA-1 ligation during transendothelial migration was recently found to drive the synthesis of intracellular complement C3, which was among the most enriched molecular signature of tissue lymphocytes. Examining the T cells from patients with LAD syndrome, the study authors observed reduced *C3* mRNA and impaired effector cytokine production (Kolev et al., 2020). Other integrins besides LFA-1 likely contribute to transendothelial migration because the double deletion of the cytoskeletal effector proteins, VASP and EVL, impaired T-cell trafficking into lipopolysaccharide-treated ear skin owing to defective integrin α4 function. Strikingly, T cells deficient in both VASP and EVL exhibited normal crawling and adhesion under shear flow but defective transendothelial migration (Estin et al., 2017).

Because T-cell entry into tissue is a critical point in their functional capability, the processes of rolling, firm adhesion, and transendothelial migration are highly influenced by inflammatory and pathologic conditions. Such stimuli modify adhesion molecule expression on both T cells and the endothelium to regulate lymphocyte entry into the skin (Capece et al., 2017; DeNucci et al., 2010; Ley and Kansas, 2004; Mahuron et al., 2020). For example, analysis of peripheral blood from both adult and pediatric patients with atopic dermatitis (AD) revealed the expansion of CLA^+^ Th2 cells, consistent with the type-2 inflammatory skew of this disease (Czarnowicki et al., 2015a, 2015b). In ex vivo transendothelial migration experiments, blood-derived T cells from patients with scleroderma exhibited significantly enhanced migratory ability compared with healthy control cells (Stummvoll et al., 2004). In support of these findings, CD4^+^ T cells from patients with scleroderma not only expressed elevated levels of CD11a (the α_L_ subunit of LFA-1) but also reduced the methylation of the CD11a promoter, indicating an epigenetically encoded propensity for increased integrin expression (Wang et al., 2014). These results suggest that in patients with skin disease, circulating T cells are likely ectopically primed for tissue entry.

#### Interstitial migration

After passing through the endothelium, T cells enter into a complex 3‒dimensional interstitial space comprising tissue cells, soluble signaling mediators, and the ECM. T cells must integrate cues from this environment to patrol the tissue and position themselves within the skin microarchitecture (Gaylo et al., 2016). Cutaneous tissue is a dynamic and high-force environment that experiences differential levels of mechanical stress across the epidermis and dermis and during tissue renewal (Biggs et al., 2020; Hsu et al., 2018). Notably, injury and inflammation precipitate the remodeling of the ECM, which influence the signals T cells receive through their adhesion receptors (Gaylo et al., 2016; Hsu et al., 2018). Consequently, T-cell motility behaviors (i.e.*,* travel speed and area of immunosurveillance) are directly dictated by the mechanical landscape within the tissue (Gaylo et al., 2016). An abnormal distribution of tissue forces is a feature of several skin disorders (Hsu et al., 2018).

T-cell subsets appear to differentially segregate in the skin. A large subset of Tregs localizes to HFs (Ali et al., 2017b; Sanchez Rodriguez et al., 2014). Effector and memory CD4^+^ T cells are also commonly observed around the HFs; however, these cells also patrol within the dermis (Collins et al., 2016; Gaylo et al., 2016; Gebhardt et al., 2011). In contrast, CD8^+^ T cells and DETCs preferentially associate with the epidermal basement membrane (BM), although in healthy human skin, a relatively large proportion of CD8s inhabit the epidermis and are capable of migrating back and forth between the dermis and epidermis (Cheuk et al., 2014; Dijkgraaf et al., 2019; Gebhardt et al., 2011; Thelen and Witherden, 2020). Interstitial motility of skin T cells was presumed to be through integrin-independent amoeboid crawling, as has been described in lymph nodes and for myeloid cells (Gaylo et al., 2016; Jacobelli et al., 2009; Lämmermann et al., 2008; Weninger et al., 2014). However, evidence suggests that at least in the context of inflammation, effector CD4^+^ T cells require integrin α_v_ to move within the dermis (Fernandes et al., 2020; Overstreet et al., 2013). Patrolling T cells use the ECM as a scaffold for interstitial migration, relying on α_v_β_1_ and α_v_β_3_ interactions with fibronectin-decorating collagen fibers (Fernandes et al., 2020; Overstreet et al., 2013). The differences between the motility observed during steady state and inflammation are likely determined by structural remodeling of the ECM (Fernandes et al., 2020; Gaylo et al., 2016; Overstreet et al., 2013; Sorokin, 2010). In this process there is also evidence of subset-driven variation because Th2 differentiation increases α_v_β_3_ expression compared with Th1 cells. Functionally, Th2 cells were observed to patrol a larger area of inflamed dermis than Th1 cells, and manipulation of α_v_β_3_ levels was sufficient to reverse this behavior (Gaylo-Moynihan et al., 2019).

Transit between the dermis and epidermis in healthy skin is likely facilitated by pores in the epithelial BM, although this has not been studied in detail (Oakford et al., 2011). Leukocyte trafficking through such structures is presumably an integrin-independent process owing to their dependency on protrusion-based contractile motility when passing through tight spaces (Jacobelli et al., 2010; Kelley et al., 2014; Lämmermann et al., 2008). Nonetheless, integrin α_1_β_1_ blockade with a mAb against α_1_ (CD49a) markedly reduced the accumulation of T cells in the epidermis of human psoriatic skin xenografts. Treatment with this antibody also mitigated otherwise spontaneous progression of asymptotic transplants toward pathology, implicating epidermal T cells as mediators of psoriasis (Conrad et al., 2007). However, a more recent study examining α_1_ knockout in murine T-cell responses to Herpes simplex virus infection found no requirement for this integrin in epidermal localization (Bromley et al., 2020). Instead, α_1_ supported CD8^+^ Trm cell persistence in the epidermis, promoted the formation of dendrite protrusions in these cells, and was directly linked to effector function through the induction of IFN-γ (Bromley et al., 2020; Cheuk et al., 2017). Given that the original blocking experiments were performed in the context of transplantation and relied on the skin from patients with psoriasis, it is possible that specific inflammatory signals lead to reorganization of the ECM and precipitate a requirement for α_1_β_1_ in dermal to epidermal transit. Integrin α_E_β_7_ is another adhesion molecule implicated in T-cell function at the epithelium. Induced on T cells by TGFβ signaling, α_E_β_7_ binds to E-cadherin, which is highly expressed on epithelial cells (Hardenberg et al., 2018; Sivasankar, 2013). Although CD103 (α_E_) was found to influence DETC dendrite formation, it did not contribute to the cellular morphology of CD8^+^ Trm cells (Schlickum et al., 2008; Zaid et al., 2017). Trm cells deficient in CD103 demonstrated increased motility within the epidermis, suggesting that this integrin acts to locally restrain (or guide) T-cell migration (Zaid et al., 2017). Consistent with this possibility, deletion of CD103 did not impact the entry of CD8^+^ Trm cells in the epidermis but was required for their continued persistence at this skin compartment (MacKay et al., 2013).

#### Cell‒cell interactions

In addition to meditating motility, adhesion molecules are inextricably linked to T-cell activation and differentiation. LFA-1, VLA-4, and α_E_β_7_ are participants in immunological synapse formation and, through engagement with ligands on antigen-presenting or target cells, modulate TCR signaling (Basu and Huse, 2017; Hammer et al., 2019; Hardenberg et al., 2018; Jankowska et al., 2018). This contribution to cell‒cell interaction is directly pertinent to CD8^+^ effector function because either LFA-1 or α_E_β_7_ is required for efficient cytotoxicity toward cancer cells (Franciszkiewicz et al. 2013; Le Floc’h et al. 2007). A B16 melanoma cell line engineered to overexpress E-cadherin was more easily eliminated by immune cells, suggesting that the stoichiometry of integrin‒cadherin interactions between T cells and their targets regulates effector activity (Shields et al., 2019). In agreement with these data, culturing CD8^+^ Trm cells on the α_1_ ligand, collagen IV, augments IFN-γ production (Cheuk et al., 2017). Skin epithelium may also be able to provide T-costimulatory signals by LFA-1. Primary human keratinocyte cultures pretreated with IFN-γ were able to activate naïve peripheral blood T cells and induce differentiation toward a Th1 and Th17 phenotype (Orlik et al., 2020). Whereas CD2 ligation is necessary for full T-cell activation, LFA-1 binding to ICAM-1 facilitates cell‒cell contact and promotes productive signaling (Orlik et al., 2020; Varga et al., 2010; Verma et al., 2016). Targeting molecular regulators of LFA-1 activation, including its inhibitor MAP4K4 and layilin, has shown potential as a strategy for the treatment of tumors and viral infection (Esen et al., 2020; Mahuron et al., 2020).

#### Recirculation

A large proportion of antigen-experienced skin T cells eventually exit the tissue through draining lymphatics to reenter blood circulation (Bromley et al., 2013; Brown et al., 2010; Collins et al., 2016; Hunter et al., 2016). Even human CD4^+^CD103^+^ Trm cells have been identified in circulation, indicating the potential for this population to be mobilized from tissues (Klicznik et al., 2019). T-cell emigration from the skin appears to be at least partially dependent on CCR7/CCL21 and appears to be restrained by S1P signaling (Bromley et al., 2013; Laidlaw et al., 2019; Ledgerwood et al., 2008). Notably, S1P receptor antagonism was found to halt lymphocyte egress by inhibiting migration across lymphatic endothelium in an LFA-1/ICAM-1– and VLA-4/VCAM-1–dependent manner (Ledgerwood et al., 2008). Tregs but not other T cells have been observed to facilitate their own egress by directly promoting the interactions with endothelial VCAM-1 (Brinkman et al., 2016). Presentation of lymphotoxin by Tregs to lymphatic endothelial cells induces VCAM-1 expression and is associated with Treg emigration to the draining lymph node (Brinkman et al., 2016; Piao et al., 2018). More recent intravital microscopy experiments revealed that T cells actively crawl through the skin afferent lymphatic capillaries before reaching larger collector vessels. This crawling is dependent on LFA-1 and is modulated by inflammation. Contact hypersensitivity increases T-cell velocity through the upregulation of ICAM-1 on lymphatic endothelial cells. Whereas VLA-4 was dispensable for T-cell movement within the lymphatics, its blockade reduced migration from inflamed skin to the draining lymph node, suggesting a contribution for this integrin to initial lymphatic entry (Teijeira et al., 2017).

#### A new horizon in T-cell adhesion

Although there is increasing evidence pointing to tissue and cellular specificity as a major driver of adhesion dynamics, many of the molecular pathways discussed earlier are broadly conserved across leukocyte lineages. Transformational advances in understanding T-cell function will come through linking cellular behavior to the biochemistry of adhesion molecules dissected in other leukocytes. For example, although we have highlighted several emerging examples of tissue specificity in T-cell migration, there is significantly stronger evidence for this concept in neutrophil tissue recruitment (Margraf et al., 2019). Contributing mechanisms, such as the existence of a β_2_ integrin bent-open headpiece conformation that impairs neutrophil adhesion as well as the reverse transendothelial migratory capability of these cells, have not been deeply examined in T cells (Burn and Alvarez, 2017; Fan et al., 2016; Margraf et al., 2019). In addition, focused efforts to understand T-cell adhesion in the context of their natural three-dimensional environment will be of the highest yield. Advances in intravital microscopy as well as organoid culture systems provide promise toward illuminating human T-cell behavior in the skin.

### Adhesion-based therapeutics in skin disease: promises and challenges

Because T-cell biology is inextricably linked to cellular adhesion, manipulating these pathways in inflammatory disorders, including in skin diseases, has long been recognized as a compelling therapeutic strategy (Figure 3). However, the fundamental importance and complexity of adhesion biology present an enormous challenge. Designing a drug to target molecules such as integrins requires a deep understanding of molecular mechanisms across multiple biological states, tissues, and cell types and against a backdrop of intrinsic regulatory layers. Nonetheless, overcoming these difficulties presents an exceptional opportunity for creating highly efficacious precision medicines.

**Figure 3:**
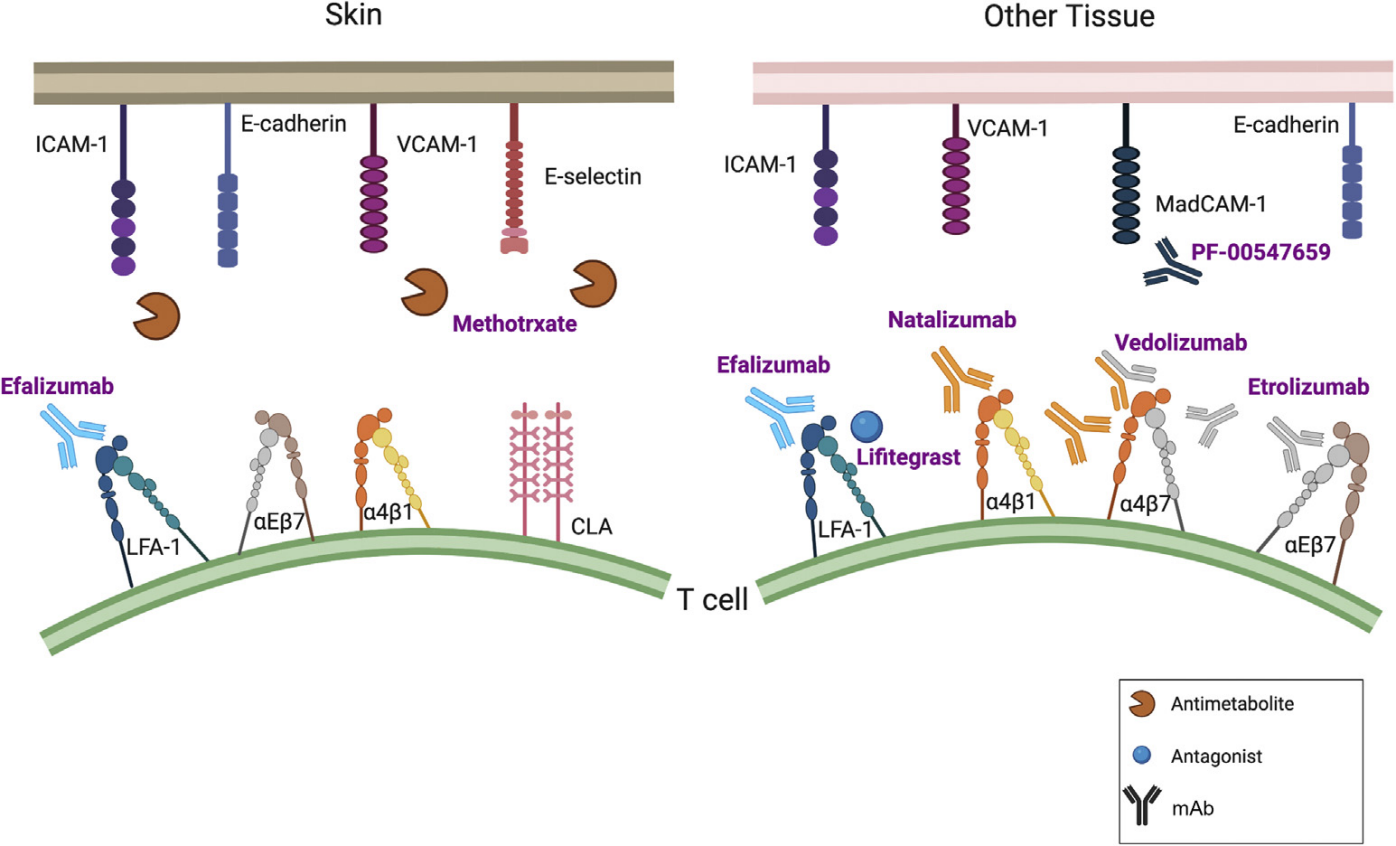
**Strategies to target adhesion molecules in disease.** Several adhesion molecule inhibitors (both small molecules and mAbs) have been developed to treat inflammatory disorders. To date, efalizumab is the only integrin-blocking therapy to have been approved for use in skin diseases, although it was subsequently withdrawn owing to severe complications. Nonetheless, these drugs illustrate potential opportunities and pitfalls in designing adhesion-based therapeutics. Whereas vedolizumab exhibits exquisite specificity by binding to a conformational epitope unique to the heterodimerization of α_4_ and β_7_ integrins, natalizumab and efalizumab encountered problems owing to the promiscuity of their integrin targets (α_4_ and α_L_, respectively). Etrolizumab, a β_7_ integrin‒blocking monoclonal in phase III clinical trials for the treatment of IBD may have relevance to skin disease given the importance of α_E_β_7_ on skin-resident T cells (Sandborn et al., 2020). One of methotrexate’s proposed mechanisms is the downregulation of selectin and immunoglobulin superfamily molecules expressed on endothelial cells. However, only limited success has been observed with a monoclonal (PF-00547659) targeting MAdCAM-1 on mucosal endothelium (Sandborn et al., 2018). IBD, inflammatory bowel disease.

Therapies targeting adhesion pathways are currently approved for the treatment of autoimmune and inflammatory diseases, including multiple sclerosis, Crohn’s disease, and UC (Alsoud et al., 2020; Ley et al., 2016). Predominantly, these approaches rely on a mAb-based blockade of integrin function, although a small molecule LFA-1 antagonist, lifitegrast, is currently approved for dry eye disease (Haber et al., 2019). Vedolizumab, which possesses a safe and effective track record in the treatment of Crohn’s disease and UC (Alsoud et al., 2020; Feagan et al., 2013), provides an illustrative example for the successful development of tissue-specific adhesion‒based therapeutics. Vedolizumab binds to a conformational epitope unique to the heterodimerization of human α_4_ and β_7_ integrins. This allows precise antagonism of α_4_β_7_ interactions with MAdCAM-1 on mucosal endothelial cells (Soler et al., 2009). Similar to the role of CLA in skin entry, MAdCAM-1 binding mediates the transmigration of T cells across the intestinal mucosa (Fedyk et al., 2012; Soler et al., 2009). Clinical studies and nonhuman primate experiments demonstrate that vedolizumab’s molecular specificity translates into targeted immunomodulation. Healthy subjects and animals treated with vedolizumab exhibited significant loss of β_7_^+^ leukocytes from the intestinal tract but not from other tissues (Fedyk et al., 2012; Milch et al., 2013).

Vedolizumab’s success in achieving a tissue-specific response contrasts with that of natalizumab, another approved anti-α_4_β_7_ monoclonal. Binding an α_4_ epitope, natalizumab, blocks both α_4_β_7_ and α_4_β_1_ (VLA-1), which is more widely expressed, including on skin-homing T cells (discussed earlier) (Fedyk et al., 2012; Ley et al., 2016). Unfortunately, this comparatively reduced specificity manifests in the potential for developing progressive multifocal leukoencephalopathy (PML), a life-threatening CNS side effect due to reactivation of the polyoma John Cunningham (JC) virus (Cortese et al., 2021; Ley et al., 2016; Milch et al., 2013). Although the exact pathogenesis of this complication remains unclear, blockade of α_4_β_1_‒VCAM-1 interaction is a likely culprit. JC virus‒reactive T cells are important contributors to viral control and disease prevention, but in contrast to vedolizumab, natalizumab treatment is associated with a reduction in CNS T-cell populations. (Cortese et al., 2021; Ley et al., 2016; Milch et al., 2013).

Efalizumab, which was briefly approved for treating psoriasis before being removed from the market owing to incidences of PML (complication risk of 1 in 400 compared with that of 1 in 1,000 for natalizumab), illustrates an additional complexity in developing integrin-based drugs (Cortese et al., 2021; Ley et al., 2016). An anti-α_L_ monoclonal, efalizumab, was expected to be highly specific for blocking LFA-1 (α_L_β_2_) because the α_L_ integrin chain only pairs with β_2_ (Ley et al., 2016; Mancuso et al., 2020). However, although efalizumab effectively reduces LFA-1 at the T-cell surface, it was also observed to cause the downregulation of α_4_β_1_ (Grönholm et al., 2016; Guttman-Yassky et al., 2008; Mancuso et al., 2020). The mechanisms underlying this response are unclear; however, outside-in signaling through LFA-1 is known to influence α_4_β_1,_ and crosstalk between these integrins is a feature of their molecular function (Grönholm et al., 2016; Kim and Hammer, 2019; Mancuso et al., 2020). In vitro experiments indicate that efalizumab can induce α_4_β_1_ without LFA-1 activation, which is a requirement for integrin crosstalk (Mancuso et al., 2020). Therefore, α_4_β_1_ downregulation may occur through additional biochemical interactions.

Although efalizumab ultimately proved disappointing, its effectiveness at reducing cutaneous inflammation is clear. A phase 3 clinical trial testing efalizumab in the treatment of psoriasis found that the majority of patients experienced at least a 50% reduction in lesion severity and area (Lebwohl et al., 2003). Several case reports and small trials have also reported success in using efalizumab to treat hypertrophic lupus erythematosus, lichen planus, and AD (Böhm and Luger, 2007; Navarini et al., 2010; Takiguchi et al., 2007). Follow-up analysis on patients with relapsing psoriasis after cessation of efalizumab observed renewed T-cell and myeloid skin infiltration in skin lesions (Johnson-Huang et al., 2012). In addition to reducing skin homing of pathogenic T-cell clones, efalizumab induces T-cell hyporesponsiveness characterized by attenuated T-cell activation and downregulation of costimulatory and TCR complex molecules (Guttman-Yassky et al., 2008; Kuschei et al., 2011). Together, these findings support the potential for targeting LFA-1‒mediated adhesion in skin disease.

An alternative approach to integrin blockade is to target selectins and their ligands. Modulation of selectin interactions required for T-cell entry into the skin may improve selectivity toward inflamed tissue because the expression of this molecule is highly sensitive to inflammatory stimuli (Ley and Kansas, 2004). Methotrexate is a synthetic folic acid analog and anti-inflammatory agent that has long been used in the treatment of inflammatory skin disorders (Shen et al., 2012). Although methotrexate has broad effects, it is interesting that at least some of its anti-inflammatory properties are due to the regulation of adhesion molecules and prevention of lymphocyte accumulation in the skin. Specifically, methotrexate treatment reduces endothelial expression of E-selectin and VCAM-1 while also downregulating CLA on T cells (Dahlman-Ghozlan et al., 2004; Johnston et al., 2005; Sigmundsdottir et al., 2004). Preclinical experiments attempting to directly target E-selectin with a blocking mAb successfully prevented human Th2-cell infiltration into human skin xenografts (Biedermann et al., 2002). Likewise, administration of a sugar derivative pan-selectin inhibitor reduced inflammation in a mouse model of allergic dermatitis (Ikegami-Kuzuhara et al., 2001). However, a clinical trial treating patients with psoriasis observed no therapeutic benefit of E-selectin blockade (Bhushan et al., 2002).

A potentially attractive therapeutic approach is targeting integrin modulating proteins. This may have the advantage of inhibiting or augmenting adhesion of specific lymphocyte subsets in specific tissues only in inflammatory or malignant contexts. As mentioned previously, the C-type lectin, layilin, modulates LFA-1 activation on CD8^+^ T cells and is only expressed on a limited number of lymphocyte populations in inflamed or malignant skin (Liu et al., 2020; Mahuron et al., 2020). Thus, selective modulation of proteins such as layilin may have a beneficial therapeutic effect with fewer adverse reactions than directly targeting integrins or their ligands.

### Conclusions and outlook

Adhesion molecules are critical mediators of T-cell accumulation and function in nonlymphoid tissues. These molecules exert functional effects almost continuously as T cells enter and traverse the skin. This breadth of activity means that adhesion mechanisms are highly complex and exhibit multiple layers of regulation and molecular redundancy. Nevertheless, recent research has uncovered several examples of tissue and cell-type specificities, such as between Th1 and Th2 cells or between Tregs and effector T cells. Small variations in how T-cell populations use adhesion molecules to interact with their local environment can result in a large influence on immune response and disease pathology. Because many inflammatory skin disorders have a strong T-cell component, modulating adhesive interactions has long been considered an attractive therapeutic strategy; yet, this approach has most likely not realized its full potential. Despite the current absence of an approved adhesion-targeting therapy for skin diseases, a detailed understanding of relevant molecular pathways has grown significantly since efalizumab was first developed. Developing drugs to target T-cell adhesion presents a formidable challenge in overcoming intrinsic molecular redundancy and functional complexity. The severe off-target complications associated with efalizumab and natalizumab reinforce this difficulty and emphasize a need to comprehensively consider epitope specificity, expression profile, and molecular regulation in designing therapeutics to modulate cellular adhesion. At the same time, doing so opens the possibility for harnessing exquisite biological precision.

## ORCIDs

Joshua M. Moreau: http://orcid.org/0000-0002-1227-1508

Victoire Gouirand: http://orcid.org/0000-0002-2666-0061

Michael D. Rosenblum: http://orcid.org/0000-0002-0462-5732

## Author Contributions

Conceptualization: JMM, VG, MDR; Investigation: JMM, VG, MDR; Methodology: JMM, VG, MDR; Project Administration: JMM, VG, MDR; Resources: JMM, VG, MDR; Software: JMM, VG, MDR; Supervision: MDR; Validation: JMM, VG, MDR; Visualization: JMM, VG, MDR; Writing - Original Draft Preparation: JMM, VG, MDR; Writing - Review and Editing: JMM, VG, MDR
